# The HTLV-1 hbz antisense gene indirectly promotes tax expression via down-regulation of p30-II mRNA

**DOI:** 10.1186/1742-4690-8-S1-A136

**Published:** 2011-06-06

**Authors:** Gunjan Choudhary, Daniel Rauch, Lee Ratner

**Affiliations:** 1 Division of Molecular Oncology, St Louis, MO, 63110, USA

## 

Human T-cell leukemia virus type 1 (HTLV-1) basic leucine zipper factor (HBZ) is transcribed from the antisense genomic DNA strand and functions differently in its RNA and protein forms. To distinguish between the roles of hbz mRNA and HBZ protein, we generated mutants in a proviral clone that specifically disrupt the hbz gene product. A proviral clone with a splice acceptor mutation that disrupts expression of the predominant hbz mRNA resulted in lower levels of tax mRNA. Heterologous hbz expression restored Tax activity in cells expressing this mutant clone. In contrast, proviral mutants that disrupt only HBZ protein did not affect levels of tax mRNA. Expression of hbz resulted in lower levels of p30II mRNA. Mutation of p30II overcame the effects of the splice acceptor mutation of hbz, and restored tax expression. Thus, there is a complex interplay of viral regulatory proteins controlling levels of HTLV-1 gene expression. The pathogenic role of HBZ alone or combined with Tax is currently under study in transgenic mice, and will be discussed.

